# Illness Expectations and Asthma Symptoms: A 6‐Month Longitudinal Study

**DOI:** 10.1111/hex.70285

**Published:** 2025-05-05

**Authors:** Eleonora Volpato, Valentina Poletti, Paolo Banfi, Andrea Bonanomi, Francesco Pagnini

**Affiliations:** ^1^ Department of Psychology Università Cattolica del Sacro Cuore Milan Italy; ^2^ IRCCS Fondazione Don Carlo Gnocchi Milan Italy; ^3^ Research Group Health Psychology University of Leuven Leuven Belgium; ^4^ Department of Statistical Science Università Cattolica del Sacro Cuore Milan Italy

**Keywords:** adherence, asthma, beliefs, illness expectations, mind–body connection

## Abstract

**Background:**

After receiving a diagnosis, individuals often develop expectations about how their condition will evolve. This cognitive framework, known as ‘Illness Expectations’ (IEs), encompasses future‐oriented beliefs regarding the course of the illness and its symptoms. In chronic conditions such as asthma, IEs may play a critical role in shaping patient‐reported outcomes and clinical markers of disease progression. This study aims to empirically evaluate the impact of IEs on asthma symptoms and respiratory function.

**Methods:**

A cohort of 310 individuals diagnosed with asthma was followed over a 6‐month period, with three assessment points. Asthma control was measured using the Asthma Control Test (ACT), while respiratory function was evaluated through forced expiratory volume in 1 s (FEV_1_) using spirometry. IEs were assessed using the validated ‘Illness Expectation Test’ (IET), which captures both explicit (conscious) and implicit (unconscious) expectations. Predictive analyses were conducted using latent growth modelling and linear regression to examine the influence of IEs on asthma symptoms and respiratory function over time.

**Results:**

People with more negative explicit IEs about their asthma reported worse symptoms over time (*β* = −0.50, SE = 0.21, *p* = 0.01). Implicit IEs were not statistically significant (*β* = −0.014, SE = 0.008, *p* = 0.09). Explicit IEs about symptom progression were also associated with changes in lung function, with more negative expectations predicting greater declines in respiratory performance (*β* = 0.51, SE = 0.11, *p* = 0.001).

**Conclusions:**

These findings suggest that IEs may be meaningfully associated with asthma outcomes, highlighting their potential relevance in understanding patient experiences and symptom perception. These results support further research into interventions targeting cognitive frameworks, with the aim of informing more personalised, patient‐centred approaches to asthma management.

**Patient or Public Contribution:**

This study was developed in response to patient‐reported challenges in asthma management, particularly around understanding and managing IEs. Patients contributed to identifying key areas of concern, and their perspectives informed the choice of outcomes and tools. While direct involvement in recruitment and dissemination was limited due to the pandemic, the study's design and focus were guided by patient priorities, with potential applications in clinical consultations and future co‐designed interventions.

**Abbreviations**
ABS: Absolute Expectations
ACT: Asthma Control Test
FEV_1_
: Forced expiratory volume in 1 s
FEV_1_/FVC%: The FEV_1_/FVC ratio, also called modified Tiffeneau–Pinelli index
FVC: Forced vital capacity
GHPS: The Good Health Practices Scale
GINA: Global Initiative for Asthma
GLMs: Generalised Linear Mediation Models
HADS: The Hospital Anxiety and Depression Scale
HADS‐A: Anxiety subscale
HADS‐D: Depression subscale
IE: Illness Expectation
IEs: Illness Expectations
IET: Illness Expectation Test
IE‐IAT: IE‐specific Implicit Association Test
IMP: Improvement/Worsening
LMMs: Linear Mixed Models
MPAS: Measures of Patient Adherence Scale
NA: Negative Affects
PEF: Peak Expiratory Flow
PSS: Perceived Stress Scale
RIG: Rigidity
SD: standard deviation
STROBE: Strengthening the Reporting of Observational Studies in Epidemiology

## 1 Introduction

### 1.1 Background/Rationale

In contemporary health research and clinical practice, there has been growing recognition of the limitations of the traditional biomedical model, which primarily emphasises biological processes. Increasingly, integrative frameworks such as the biopsychosocial model [1] and mind–body paradigms [2] have been embraced to account for the complex interplay between biological, psychological and social factors in health and disease. Specifically, the biopsychosocial paradigm underscores the intricate interplay between psychological and physiological systems, challenging the traditional dichotomy of mind and body. Stressful emotional states can adversely affect immunity [3, 4], wound healing [5], vaccination efficacy [6, 7], as well as respiratory health [8, 9] through complex neuroendocrine and immune mechanisms [3, 5, 7, 8]. This underscores the multifaceted influence of psychological factors on health outcomes, emphasising the significance of cognitive processes in shaping individual health trajectories.

Beliefs significantly influence health outcomes through multiple pathways. Research highlights their critical role in guiding health‐related decisions [10] and the powerful impact of anticipatory expectations on outcomes. Similar to findings in pain management, where patient awareness of treatment can alter pain perception [11], studies in respiratory diseases have demonstrated that cognitive factors—such as emotional state and awareness—can significantly influence symptom perception [9].

Additionally, perceiving a sense of purpose strongly correlates with better health outcomes, including lower rates of mortality, improved cardiovascular health and enhanced mental well‐being. These effects are thought to arise through mechanisms such as reduced stress reactivity, healthier lifestyle behaviours and greater resilience to adversity [12, 13, 14], further attesting to the intricate interplay between psychological factors and physical well‐being. The placebo effect further illustrates how beliefs and expectations modulate physiological responses [15, 16]. Notably, the anticipation of change, induced by a primer (e.g., a sham pill), can lead to tangible physical alterations, suggesting that beliefs about a diagnosis may directly influence its progression [17]. A substantial body of literature delves into placebo and nocebo effects, highlighting their role in shaping health outcomes [18, 19, 20]. However, it is important to note that the evidence is not without critique. Some researchers argue that the effects attributed to placebos may be overestimated due to methodological challenges, such as insufficient control groups, reporting bias and difficulty distinguishing placebo effects from natural symptom variation or regression to the mean [21, 22]. Despite these critiques, many studies demonstrate that patient expectations, even in controlled settings, can significantly influence subjective and objective health outcomes.

Illness Expectation (IE) [23] refers to the cognitive framework constructed by individuals diagnosed with a chronic illness, shaping anticipated characteristics of disease progression and future‐oriented beliefs about symptoms. This framework includes individual abilities, cognitive and emotional processing based on personal history, and knowledge about the diagnosis and course of the condition, influenced by information received [23]. Not all individual's IEs will be consciously recognised by them; similar to other expectation types [24], some IEs will appear as explicit (conscious) cognitions, while others will remain implicit, such as automatic associations and unconscious beliefs [23]. IE can exhibit varying degrees of rigidity, spanning from subtle expectations to a highly entrenched conception of the expected disease progression [23]. IE is reflective of an individual characteristic, and persons with identical diagnoses and exposure to comparable information may harbour diverse IEs. The information received, particularly in the context of medical treatments, including placebos [25], can significantly influence IEs.

The IE model posits that the impact of mindsets—the underlying cognitive frameworks or belief systems that individuals hold about their health, illness and the effectiveness of treatments—on the body can occur either through behavioural modifications (such as adherence and lifestyle changes) [26] or through a non‐behavioural pathway (physiological or psychological changes that occur independently of observable behavioural modifications) [27]. Applying this model, our investigation centres on asthma, a debilitating respiratory condition characterised by airway hyperresponsiveness, airflow obstruction and chronic inflammation [28]. Globally, the prevalence of doctor‐diagnosed asthma is estimated at 4.3%, while clinical or treated asthma has been reported at 4.5% and 8.6%, respectively, in different adult populations. These rates vary widely across countries, with a 21‐fold difference observed among the 70 countries included in a large international study [29]. This disorder's unpredictable character can cause a range of psychological reactions, including worry, anxiety and fear [30, 31]. Thus, understanding the illness can provide a feeling of control, which in turn can have an empowering effect [32]. Although illness perception and its emotional impact on treatment adherence [33, 34], responsibility [35, 36] and self‐management [37, 38] have been explored, research on the role of IEs in asthma remains limited. Previous studies have focused only on explicit, conscious expectations [33, 39, 40, 41, 42], neglecting the influence of implicit, automatic beliefs on disease‐related outcomes.

This study addresses the gap in understanding the role of IEs in asthma by examining both implicit and explicit IEs and their predictive validity for disease‐related outcomes. IEs refer to patients' beliefs and perceptions about their illness, including expectations regarding symptom progression, treatment efficacy and overall health outcomes. These expectations can influence disease management, adherence to treatment and quality of life. Explicit IEs are measured through direct self‐report questionnaires that assess individuals' conscious beliefs about their illness, while implicit IEs are assessed using indirect methods, such as reaction time tasks or Implicit Association Tests (IATs), that capture automatic, unconscious associations with the disease [39]. The aim of this study is not to develop a new tool but to apply existing methodologies to assess these two domains of IEs and investigate their relationship with disease outcomes over time. By incorporating both implicit and explicit dimensions of IEs, this study provides novel insights into how cognitive frameworks shape the trajectory of asthma. Practically, it lays the groundwork for personalised interventions that target maladaptive expectations, which could improve asthma management and patient outcomes. The findings also offer valuable insights for future research, particularly in the broader field of health psychology, where IEs play a role in managing chronic diseases.

### 1.2 Objectives

The *primary objective* of this study was to develop and test a model assessing the relationship between IEs and disease‐related outcomes, including self‐reported asthma symptoms and clinical parameters (such as respiratory function). The model aimed to explore both direct and indirect effects of IEs on disease‐related outcomes, with a specific focus on self‐reported asthma symptoms (ACT) and respiratory function (FVC). The study further sought to investigate how behavioural changes might mediate the relationship between IEs and disease outcomes, considering various potential mediators and confounders.

*Secondary objectives* included:

- Exploring the potential mediating role of disease behaviours (e.g., adherence to treatment and lifestyle factors) in the relationship between IEs and disease outcomes.
- Investigating the influence of negative emotions (e.g., stress, anxiety and depression) on disease outcomes, with a focus on understanding their potential role in influencing asthma‐related symptoms and inflammation.

## 2 Materials and Methods

The project was carried out in accordance with the Helsinki Declaration, and the protocol was authorised by the Università Cattolica del Sacro Cuore's Ethics Committee (cod. 35–18, 21/12/2018), in Milan, Italy, and by the IRCCS Fondazione Don Carlo Gnocchi's Ethics Committee (cod. 14/2019/CE_FdG/FC/SA, 17/04/2019). In accordance with Italian Law 196/2003 on Privacy and Safeguarding of Sensitive Data and the GDPR of the European Union 2016/679, the consent form was shared with participants before the interview. The dissemination of the participants' anonymised responses was permitted with their informed consent. In accordance with the Strengthening the Reporting of Observational Studies in Epidemiology (STROBE) guidelines [43], we have adhered to the recommendations for reporting observational research (Supporting Information 1).

### 2.1 Study Design, Time and Setting

A longitudinal cohort study was undertaken by enlisting potential participants from the outpatient clinics of the Heart‐Respiratory Rehabilitation Unit at IRCCS Fondazione Don Carlo Gnocchi, Milan, Italy. Moreover, this initiative received support from the FederAsma e Allergie OdV Network, a voluntary organisation that, since its establishment in 1994, has united Italian patient associations dedicated to advancing the battle against respiratory and allergic‐atopic diseases. Patient and public involvement was integrated throughout the study. Individuals with asthma contributed to the development of the IEs tool by providing input during the design phase, helping refine item content and wording. They also advised on recruitment strategies and contributed to dissemination planning to ensure the findings are accessible and relevant to the patient community.

The study was conducted over the period from 31 January 2021 to 1 February 2022. Each participant underwent an in‐depth medical and psychological assessment on three occasions, spaced 3 months apart, denoted as T0 (Baseline), T1 (3 months) and T2 (6 months).

### 2.2 Participants

#### 2.2.1 Inclusion and Exclusion Criteria

Participants meeting the specified criteria were enrolled in the study if they were 18 years or older; participants were recruited following a referral from physicians specialising in pulmonology. All participants had been previously diagnosed with asthma by a pulmonologist according to the Global Initiative for Asthma (GINA) guidelines [28]. Eligibility for the study required a certified asthma diagnosis from a specialist before enrolment. Asthma diagnosis was based on standard clinical criteria, including lung function tests such as spirometry, fractional exhaled nitric oxide (FeNO) measurement and peak flow monitoring, in accordance with international guidelines. Moreover, they were included if they had experienced symptoms for a duration of at least 1 year; they spoke Italian; without a mental health condition (e.g., clinically diagnosed psychiatric disorders, such as severe depression, anxiety or other cognitive disorders) based on available medical records, that may interfere with the participants' ability to comprehend or engage with study procedures; and they had the physical capability to use a computer.

#### 2.2.2 Recruitment and Sample Size

The recruitment phase proceeded on a rolling basis, but the onset of the pandemic significantly impeded its progress. The pandemic had dual ramifications for the study: firstly, the use of spirometers was either prohibited or discouraged for a considerable part of 2020‐21 due to the risk of potential SARS‐CoV‐2 transmission; secondly, psychological factors related to the pandemic may have acted as uncontrolled confounders [44], exerting a notable influence on the study outcomes. Consequently, data collection commenced in early 2021.

The sample size calculation was performed using the PAMLj package in Jamovi, based on the four most comprehensive models employed. These models assessed the relationship between various factors (such as Absolute Expectations (ABS), Improvement/Worsening (IMP) and psychological variables) and disease‐related outcomes [Asthma Control Test (ACT) and forced vital capacity (FVC)), as presented in Table 2. In relation to the models to be applied, with an effect size of 0.06, a statistical power of 0.95 and a significance level of 0.05 were adopted (see, e.g., [45]), and for nine predictors for the outcome, the estimated sample size was 200 participants [45]. Despite the initial expectation of enrolling 200 participants in the cohort, we successfully recruited a larger sample size. This expanded participant pool facilitated more nuanced analyses and bolstered the statistical power of the study. The Generalised Linear Mediation Models (GLM) presented subsequently were estimated on a reduced sample size due to dropout. However, with the final sample size, these models (Figure 3), which involve fewer relationships to estimate and fewer variables, ensure a minimum statistical power greater than 0.8 at a significance level of *α* = 0.05.

### 2.3 Procedures

Participants were identified through outreach efforts by the clinical centre (IRCCS Fondazione Don Carlo Gnocchi, Milan, Italy), which included personal invitations sent by healthcare providers, phone calls and information sessions held at the centre whenever possible. Additionally, specialised newsletters disseminated by the patients' association provided study details and invited participation from a broader network of individuals with asthma. Following verification of their eligibility, participants underwent assessment, facilitated, when possible, within pandemic restrictions, by a pulmonologist or a respiratory physiotherapist using a spirometer. Each medical evaluation took about 15 min.

Subsequently, participants engaged in the psychological assessment battery via computer, either at the hospital or from home, using the Qualtrics platform (Qualtrics, Provo, Utah, the United States). Each psychological assessment was administered by a competent Psychologist under the supervision of an experienced researcher with expertise in administration. On average, the assessment duration was 45 min. Follow‐ups occurred at 3 and 6 months for the second and third assessments, respectively, and involved the same processes as the initial assessment. Participants were contacted via phone calls, emails and newsletters from the patients' association to schedule these assessments. As an incentive, participants voluntarily opted to receive a €5 Amazon gift card at each assessment.

#### 2.3.1 Outcomes, Data Sources/Measurement

*Target variables* in the study encompass both objective and self‐reported disease‐related outcomes. The assessment included the medical evaluation, which comprised the spirometry measuring FVC and the ACT [46]. Spirometry provides an objective measure of lung function, while the ACT is a validated and reliable tool for assessing asthma control and symptom severity in both clinical practice and research settings. The ACT is recommended by global guidelines for asthma management [47, 48].

*Predictors* in this study encompassed the assessment of IEs using the Illness Expectation Test (IET)—a tool developed during the initial phase of this project [39]. This test facilitates a reliable measurement of IEs in individuals with asthma, encompassing both explicit and implicit expectations [39, 49].

Explicit expectations were evaluated through a 25‐item self‐reported questionnaire, categorising responses into three distinct factors:

- *Absolute expectations*: projecting the anticipated future state of symptoms and health.
- *Improvement/Worsening*: gauging expectations regarding changes in symptoms and health compared to the present day.
- *Rigidity*: assessing the degree to which IEs are fixed or resistant to change, the degree of rigidity, which may hinder the patient's ability to adapt to evolving disease experiences and management strategies.

The scale demonstrated robust psychometric properties, including strong internal consistency (the Cronbach's *α* values for each subscale were, respectively, 0.916, 0.964 and 0.578) and excellent construct validity. Implicit assessment was conducted using a modified version of the IAT, which exhibited a moderate correlation with explicit factors, but did not demonstrate significant test–retest reliability (*r* = 0.254, *p* = 0.167) [39, 49].

*Confounders−Negative Affects (NA)* were represented as follows:

- The Perceived Stress Scale (PSS) [50] is a 14‐item self‐report tool widely utilised to gauge the overall perception of stress in daily life. Responses range from ‘never’ to ‘very often’ on a 5‐point scale. The PSS demonstrates good reliability, with reported Cronbach's *α* values ranging from 0.74 to 0.91 across various studies, and exhibits strong correlations with measures of life events stress and social anxiety [51, 52].
- The Hospital Anxiety and Depression Scale (HADS) [53] comprises 14 items rated on a 4‐point Likert scale (range 0–3). Specifically designed for screening depression and anxiety in medical patients over the past week, the scale includes a 7‐item depression subscale (HADS‐D) and a 7‐item anxiety subscale (HADS‐A). Each subscale score is the sum of the respective seven items. Importantly, the scale primarily includes items unrelated to somatic symptoms of depression and anxiety, thereby mitigating potential overlaps with illness‐related symptoms.

*Confounders—Illness Behaviours* were detected as follows:

- The Good Health Practices Scale (GHPS) [54] is a 16‐item questionnaire derived from the original and widely utilised Wellness Maintenance within the Health Behaviour Checklist [55]. Distinguished from the original scale, it offers broader coverage of health behaviours, enhanced psychometric properties and superior predictive validity concerning actual behaviours and physiological changes. Respondents express the extent to which each item is typical for them on a 5‐point scale, with items presented in the first person [54, 55].
- The Measures of Patient Adherence Scale (MPAS) stands out as one of the most frequently employed tools for assessing treatment adherence in individuals with chronic diseases [56]. Comprising 5 items, it provides a comprehensive indication of patient adherence by prompting subjects to report how frequently certain behaviours were true for them over the past 4 weeks on a 6‐point Likert scale. The scale demonstrates strong psychometric properties, with reported Cronbach's *α* values exceeding 0.80 and significant correlations with objective measures of adherence [57].

### 2.4 Statistical Analysis

Baseline characteristics and outcomes were presented using mean and standard deviation (SD) for continuous variables, while frequency and percentage were employed for categorical variables. The correlations between variables were assessed utilising the Pearson correlation coefficient (*r*) with 95% Cls.

To explore the association between predictors (ABS, IMP and IAT) and outcomes (ACT and FVC), Linear Mixed Models (LMMs) and GLMs were implemented. LMMs were selected due to their ability to account for repeated measures data, incorporating both fixed and random effects to address within‐subject correlation and variability over time. GLM was chosen to evaluate mediation effects, providing flexibility in modelling continuous and categorical variables while testing indirect effects. The assumptions of both models were assessed. For LMM, normality of residuals was examined using Q–Q plots, while homoscedasticity and absence of multicollinearity were verified through scatterplots and Variance Inflation Factor (VIF) analysis, respectively. For GLM, the linearity of relationships between predictors and outcomes, as well as the independence of errors, was checked. Both approaches ensure robust modelling of the complex relationships in longitudinal and mediation contexts. Specifically, the GLM were used to test the following models:

– *Model 1*: Model 1 assessed the impact of explicit IEs (Absolute Expectations) at T0 as predictors of symptom control (ACT) at T1 (Dependent Variable) over time, while accounting for potential confounding variables, including negative factors (stress, anxiety and depression) and positive factors (GHP and adherence). We acknowledge that some of these variables, particularly adherence and stress, could act as mediators rather than pure confounders, reflecting pathways through which illness expectations influence symptom control. To address this, we conducted a post hoc mediation analysis using GLM. This allowed us to disentangle direct effects of IEs on symptom control from indirect effects mediated through these variables, ensuring a more nuanced understanding of the relationships between predictors, mediators and outcomes.
– *Model 2*: Investigating the influence of implicit IEs (IAT) at T0 as predictors on symptom control (ACT) at T1 over time, while controlling for similar confounders as in Model 1.
– *Model 3*: Exploring the effect of explicit IEs (‘Improvement/Worsening’) at T0 as predictors on the change in symptomatology control between T0 and T1 (DeltaACT) over time, while controlling for similar confounders as in Model 1.

Similar models were applied to the data from T2 to verify the relationships and structure observed in the earlier analysis.

A random effect was incorporated into the model to account for individual variability over time. All models were adjusted for confounders outlined in the theoretical framework [23], including the Rigidity factor from the IET. A significance level of 0.05 was adopted. We examined dropout patterns by comparing baseline, socio‐demographic characteristics of participants who completed the study versus those who dropped out. Specifically, we used *χ* ^2^ tests for categorical variables (e.g., gender) and independent *t*‐tests for continuous variables (e.g., age). The statistical analyses were carried out using Jamovi software (version 2.3.28 solid).

## 3 Results

### 3.1 Participants

**Figure 1:**
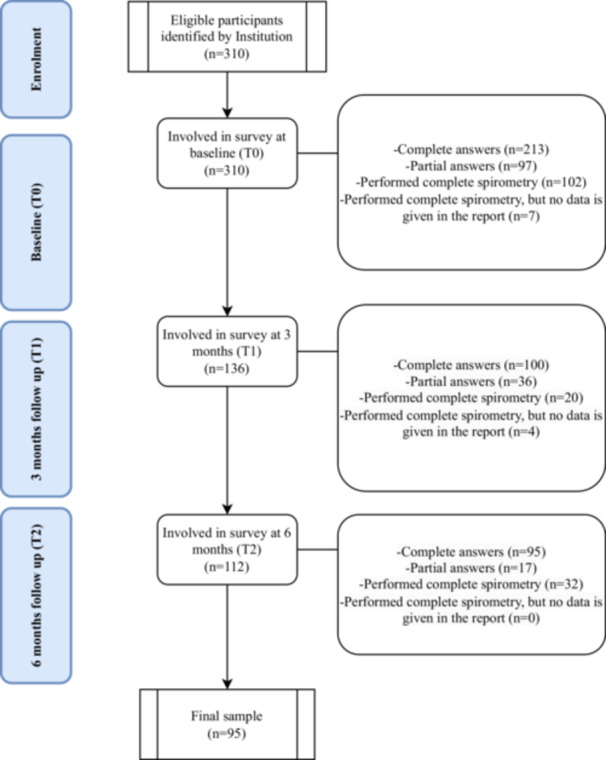
Flow chart of the sampling procedure.

A total of 310 participants initially completed the survey, with 162 identified as eligible at the Heart‐Respiratory Rehabilitation Unit at IRCCS Fondazione Don Carlo Gnocchi, in Milan, Italy, and an additional 148 recruited through the support of FederAsma e Allergie OdV Network. Among the participants, 189 were female, constituting 62.6% of the total, with a mean age of 40.81 (SD = 16.29) (Table 1). Recruitment and spirometry testing were impacted by regulatory restrictions during the pandemic, particularly in 2021 [58], as spirometry is considered an aerosol‐generating procedure. These disruptions led to a dropout rate of 69.35% over the observation period, as shown in Figure 1. A comparison of baseline socio‐demographic characteristics between participants who completed the study and those who dropped out revealed no significant differences in key variables, suggesting that dropout was not systematically related to these factors. In constructing the GLMs, we excluded variables with over 30% missing data, including spirometry measures disrupted by the SARS‐CoV‐2 pandemic. Variables were selected based on theoretical relevance and data completeness to minimise bias.

**Table 1:** Baseline characteristics of the sample.

Type of measure	Baseline (T0)	
	*N*	310
Socio‐anagraphic data	Age (M, SD)	40.81 (16.29)
	Female (%)	189 (62.60)
Marital status		
	Single (*n*, %)	99 (32.90)
	Widow/widower (*n*, %)	8 (2.58)
	Married (*n*, %)	132 (43.90)
	Separated (*n*, %)	9 (3)
	Divorced (*n*, %)	8 (2.70)
	Other (i.e., boyfriend/cohabitant…)	45 (14.51)
	No answer to this field (*n*, %)	9 (2.90)
Educational qualification		
	Primary school (*n*, %)	1 (0.30)
	Lower secondary school (*n*, %)	31 (10.30)
	Secondary school (*n*, %)	134 (44.4)
	Bachelor's degree (*n*, %)	43 (14.20)
	Master's degree (*n*, %)	67 (22.20)
	Other specialisations (e.g., Master's and Doctorate) (*n*, %)	24 (7.90)
	No answer to this field (*n*, %)	10 (3.22)
Employment status		
	Employed (*n*, %)	137 (45.40)
	Freelancer (*n*, %)	43 (14.20)
	Unemployed (*n*, %)	10 (3.30)
	Student (*n*, %)	51 (16.90)
	Retired (*n*, %)	39 (12.9)
	Other (*n*, %)	22 (7.3)
	No answer to this field (*n*, %)	8 (2.60)
	Age at disease onset (M, SD)	19.7 (16.90)
Lung function and clinical measures	FVC (M, SD)	3.91 (0.99)
	FEV_1_ (M, SD)	4.34 (10.70)
	FEV_1_/FVC% (M, SD)	82.2 (10.90)
	PEF (M, SD)	7.92 (1.84)
	ACT (M, SD)	18.67 (4.95)
Psychological measures	ABS (M, SD)	2.85 (0.76)
	RIG (M, SD)	5.09 (1.20)
	IMP (M, SD)	3.82 (0.73)
	IAT‐IE (M, SD)	0.55 (0.39)
	PSS (M, SD)	19.32 (7.40)
	HADS‐Anxiety (M, SD)	8.62 (3.30)
	HADS‐Depression (M, SD)	6.32 (2.97)
	GHP (M, SD)	58.38 (12.74)
	MPA (M, SD)	13.22 (3.57)

Abbreviations: ABS = Absolute Expectations, ACT = Asthma Control Test, FEV_1_ = forced expiratory volume in 1 s, FEV_1_/FVC% = The FEV_1_/FVC ratio, also called modified Tiffeneau–Pinelli index, FVC = forced vital capacity, GHP = Good Health Practices, HADS‐A = HADS‐Anxiety, HADS‐D = HADS‐Depression, IAT = Implicit Association Test—Illness Expectations, IMP = Improvement/Worsening, M = mean, MPA = Measures of Patient Adherence, PEF = Peak Expiratory Flow, PSS = Perceived Stress Scale, RIG = Rigidity, SD = standard deviation.

**Figure 2:**
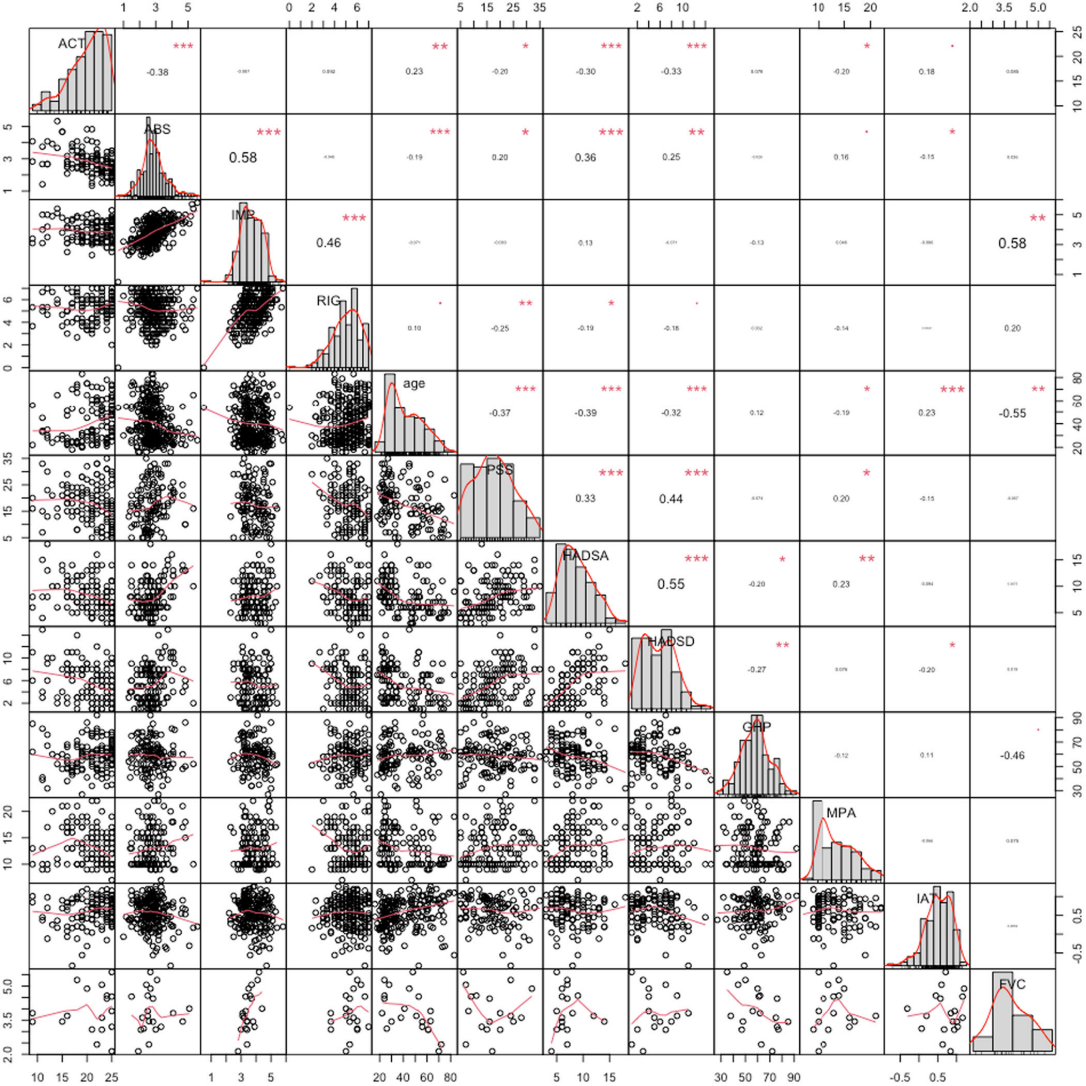
Correlation plot among all variables. Abbreviations: ABS = Absolute Expectations, ACT = Asthma Control Test, FVC = forced vital capacity, GHP = Good Health Practices, HADS‐A = HADS‐Anxiety, HADS‐D = HADS‐Depression, IAT = IAT‐IE, IMP = Improvement/Worsening, MPA = Measures of Patient Adherence, PSS = Perceived Stress Scale, RIG = Rigidity. Sig. = 0.05; *0.01; **0.001; ***< 0.001.

Figure 2 illustrates the correlation plot among all variables (Figure 2).

### 3.2 The Role of IEs in Predicting Disease‐Related Outcomes

Significant predictions were established by ABS for both ACT and FVC, even when the confounding influence of emotional factors and behaviours associated with illness was considered (*β* = −0.50, SE = 0.21, *p* = 0.01). Table 2 reports the model results.

**Table 2:** Prediction models.

		Estimate (*β*)	SE	*t* value	*p*
Prediction model of Absolute Expectations (ABS) for Asthma Control Test (ACT)	(Intercept)	32.64369	3.054	10.68883	< 0.001*
	ABS	−1.13365	0.37222	−3.04567	0.00268*
	Age	0.01535	0.02146	0.71514	0.47547
	Sex	−1.29394	0.72747	−1.77868	0.07703
	RIG	−0.37587	0.25224	−1.49012	0.13799
	PSS	0.01521	0.04034	0.37692	0.70669
	HADS‐A	−0.10054	0.1106	−0.90912	0.36454
	HADS‐D	−0.30896	0.10934	−2.82561	0.00527
	GHP	−0.0193	0.02519	−0.76646	0.44443
	MPA	−0.16552	0.07729	−2.14166	0.0336
Prediction model of Absolute Expectations (ABS) for forced vital capacity (FVC)		**Estimate (*β*)**	**SE**	***t* value**	***p***
	(Intercept)	8.66203	1.10364	7.84857	< 0.001*
	ABS	−0.34858	0.16613	−2.09826	0.04471
	Age	−0.03382	0.00712	−4.74999	< 0.001*
	Sex	−1.38404	0.20305	−6.8161	< 0.001*
	RIG	0.08466	0.08756	0.96686	0.34161
	PSS	0.0036	0.01666	0.21634	0.83023
	HADS‐A	0.09174	0.05384	1.7039	0.09909
	HADS‐D	−0.00394	0.04426	−0.0891	0.92961
	GHP	0.00146	0.00948	0.15365	0.87895
	MPA	−0.10174	0.03707	−2.74437	0.01029
Prediction model of Improvement/Worsening (IMP) factor for Asthma Control Test (ACT)		**Estimate (*β*)**	**SE**	***t* value**	***p***
	(Intercept)	32.09067	3.14184	10.21398	< 0.001*
	IMP	−0.77508	0.40104	−1.93268	0.05489
	Age	0.01902	0.02214	0.8593	0.39135
	Sex	−1.51645	0.7485	−2.02599	0.04428
	RIG	−0.10215	0.28329	−0.36059	0.71884
	PSS	0.0176	0.04069	0.43261	0.66583
	HADS‐A	−0.12267	0.11171	−1.09813	0.27365
	HADS‐D	−0.34484	0.1108	−3.11243	0.00217*
	GHP	−0.02722	0.02546	−1.06911	0.28649
	MPA	−0.17102	0.07783	−2.19738	0.02931
Prediction model of Improvement/Worsening (IMP) for forced vital capacity (FVC)		**Estimate (*β*)**	**SE**	***t* value**	***p***
	(Intercept)	8.32001	1.16687	7.13017	< 0.001*
	IMP	−0.16583	0.16754	−0.98976	0.33048
	Age	−0.02838	0.00681	−4.16676	0.00025*
	Sex	−1.44327	0.21466	−6.72368	< 0.001*
	RIG	0.1558	0.11115	1.40164	0.17164
	PSS	0.00604	0.01762	0.34276	0.73425
	HADS‐A	0.05549	0.05425	1.02277	0.31487
	HADS‐D	0.01239	0.04652	0.26641	0.79181
	GHP	−0.00407	0.00946	−0.42986	0.67048
	MPA	−0.1073	0.04178	−2.56809	0.01564

*Notes:* The table presents four prediction models assessing the relationship between various factors (such as Absolute Expectations [ABS], Improvement/Worsening [IMP] and psychological variables) and disease‐related outcomes (Asthma Control Test [ACT] and forced vital capacity [FVC])—ABS for ACT: ABS negatively predicted ACT scores; ABS for FVC: ABS was negatively associated with FVC, with age and sex also showing significant associations; IMP for ACT: IMP had a negative effect on ACT scores, with significant contributions from HADS‐D; IMP for FVC: IMP showed no significant relationship with FVC, but age and sex were significant predictors. * indicates statistically significant value.

Abbreviations: ABS = Absolute Expectations, GHP = Good Health Practices, HADS‐A = HADS‐ Anxiety, HADS‐D = HADS‐Depression, MPA = Measures of Patient Adherence, PSS = Perceived Stress Scale, RIG = Rigidity.

Even after accounting for confounders, the IMP factor strongly predicted ACT scores; however, it did not predict FVC values over time. Significant relationships with none of the disease‐related outcomes were predicted by the implicit expectations.

### 3.3 A Specific Focus on the Influence of ABS in a Mediation Model on Medical Variables

**Figure 3:**
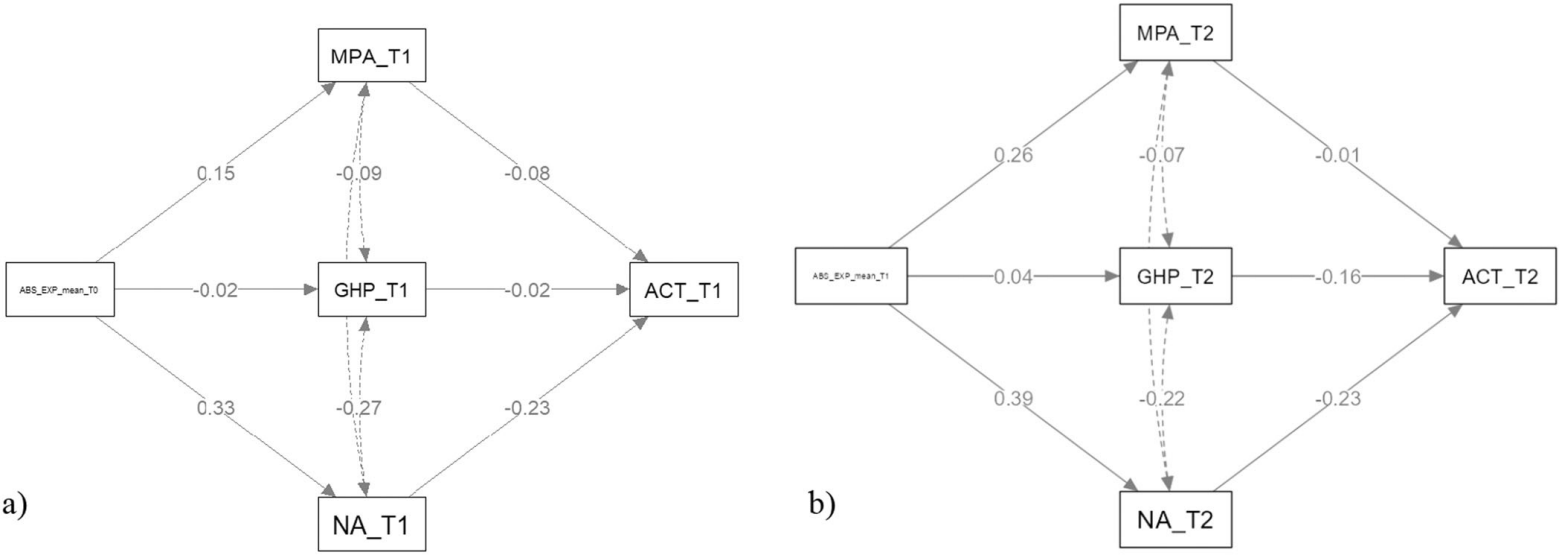
Path of the mediation model across the whole sample using Absolute Expectations (ABS) as predictors and the Asthma Control Test (ACT) as the medical outcome. *Notes:* (a) ABS_EXP_mean_T0 = Absolute Expectations as detected at T0; GHP = Good Health Practices as detected at T1; MPA = Measures of Patient Adherence as detected at T1; NA = Negative Affects (stress, anxiety and depression) as detected at T1; ACT = Asthma Control Test as detected at T1. (b) ABS_EXP_mean_T1 = Absolute Expectations as detected at T1; GHP = Good Health Practices as detected at T2; MPA = Measures of Patient Adherence as detected at T2; NA = Negative Affects (stress, anxiety and depression) as detected at T2; ACT = Asthma Control Test as detected at T2.

**Figure 4:**
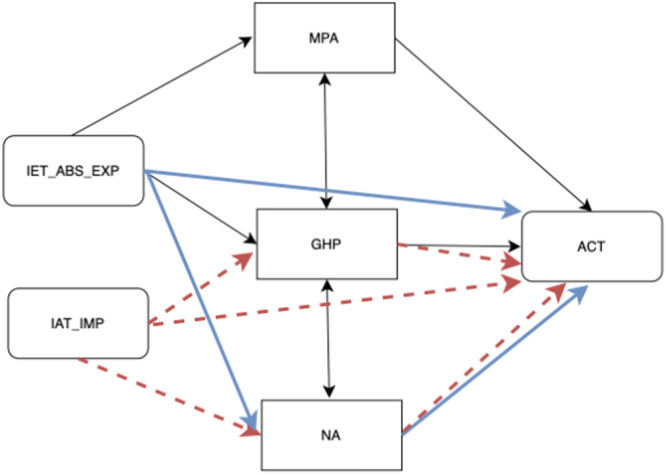
Visualisation of the main impact patterns of Explicit and Implicit Illness Expectations. *Notes:* IET_ABS_EXP = Absolute Expectations, subscale of the explicit IEs; GHP = Good Health Practices; MPA = Measures of Patient Adherence; NA = Negative Affects (stress, anxiety and depression); ACT = Asthma Control Test; IAT_IMP = Implicit Apperception Test, implicit IEs. Blue and solid arrows indicate the significant patterns of explicit IEs. Red and dashed arrows, on the other hand, indicate the non‐significant patterns of implicit IEs.

Figures 3 and 4 illustrate the path of the mediation model, examining the role of ABS as predictors and the ACT as the disease‐related outcome. The model includes GHP, MPA and NA at T1 as mediators. Path coefficients indicate the relationships between variables, with direct and indirect effects highlighting the interplay among expectations, adherence, affect and asthma control. The results reported a significant direct effect of ABS of illness on ACT and thus on the symptomatology reported by participants at T1 (*β* = −0.287; *p* = 0.001) as well as at T2 (*β* = −0.336; *p* = 0.003). The effects of ABS on ACT mediated by NA, such as stress, anxiety and depression, were also significant at T1 (*β* = −2.064; *p* = 0.039), indicating that as these variables increase, there is a worsening of the impact of ABS on symptomatology. The latter effect, however, does not occur at T2 (*β* = −0.092; *p* = 0.082). Furthermore, the direct effect of ABS on the *Δ* between ACT measured at T1 and ACT measured at T2 was not significant (*β* = 0.058; *p* = 0.562).

Table 2 shows the path values.

The results report no significant direct effect of Implicit Expectations (IAT) of illness on ACT and thus on the symptoms reported by the participants (*β* = 0.131; *p* = 0.156). The effects of IAT on ACT mediated by NA, that is, by stress, anxiety and depression (*β* = 0.048; *p* = 0.144) as well as by adopted behaviour (GHP) (*β* = −0.007; *p* = 0.604) and by adherence to therapy (MPA) (*β* = 0.007; *p* = 0.538) were also non‐significant.

Lastly, the findings reveal no statistically significant direct effect of IMP of the illness on the change in ACT scores between the considered time points, and consequently, on the alterations in reported symptoms by the participants (*β* = −0.144; *p* = 0.121). The impact of IMP on the change in ACT scores, mediated by NA, encompassing stress, anxiety and depression (*β* = 0.000; *p* = 0.921), as well as by adopted behaviours (GHP) (*β* = 0.000; *p* = 0.986) and medication adherence (MPA) (*β* = 0.002; *p* = 0.741), also yielded non‐significant effects (Table 3).

**Table 3:** Total, direct and indirect effects of GLM models.

Model 1								
Indirect and total effects								
				95% CI (a)				
Type	Effect	Estimate	SE	Lower	Upper	*β*	*z*	*p*
Indirect	ABS_EXP_mean_T0 ⇒ NA_T1 ⇒ ACT_T1	−0.396	0.192	−0.771	−0.020	−0.077	−2.064	0.039
	ABS_EXP_mean_T0 ⇒ GHP_T1 ⇒ ACT_T1	0.003	0.015	−0.027	0.032	0.001	0.179	0.858
	ABS_EXP_mean_T0 ⇒ MPA_T1 ⇒ ACT_T1	−0.063	0.078	−0.216	0.090	−0.012	−0.811	0.418
Component	ABS_EXP_mean_T0 ⇒ NA_T1	0.421	0.111	0.203	0.639	0.334	3.784	< 0.001
	NA_T1 ⇒ ACT_T1	−0.941	0.382	−1.689	−0.192	−0.231	−2.462	0.014
	ABS_EXP_mean_T0 ⇒ GHP_T1	−0.371	1.458	−3.229	2.487	−0.024	−0.255	0.799
	GHP_T1 ⇒ ACT_T1	−0.007	0.029	−0.064	0.049	−0.022	−0.252	0.801
	ABS_EXP_mean_T0 ⇒ MPA_T1	0.695	0.418	−0.124	1.515	0.154	1.662	0.096
	MPA_T1 ⇒ ACT_T1	−0.091	0.098	−0.283	0.101	−0.080	−0.929	0.353
Direct	ABS_EXP_mean_T0 ⇒ ACT_T1	−1.471	0.460	−2.373	−0.570	−0.287	−3.199	0.001
Total	ABS_EXP_mean_T0 ⇒ ACT_T1	−1.962	0.438	−2.820	−1.104	−0.377	−4.482	< 0.001

Indirect and total effects								
				95% CI (a)				
Type	Effect	Estimate	SE	Lower	Upper	*β*	*z*	*p*
Indirect	ABS_EXP_mean_T0 ⇒ NA_T1 ⇒ ACT_T1	−0.396	0.192	−0.771	−0.020	−0.077	−2.064	0.039
	ABS_EXP_mean_T0 ⇒ GHP_T1 ⇒ ACT_T1	0.003	0.015	−0.027	0.032	0.001	0.179	0.858
	ABS_EXP_mean_T0 ⇒ MPA_T1 ⇒ ACT_T1	−0.063	0.078	−0.216	0.090	−0.012	−0.811	0.418
Component	ABS_EXP_mean_T0 ⇒ NA_T1	0.421	0.111	0.203	0.639	0.334	3.784	< 0.001
	NA_T1 ⇒ ACT_T1	−0.941	0.382	−1.689	−0.192	−0.231	−2.462	0.014
	ABS_EXP_mean_T0 ⇒ GHP_T1	−0.371	1.458	−3.229	2.487	−0.024	−0.255	0.799
	GHP_T1 ⇒ ACT_T1	−0.007	0.029	−0.064	0.049	−0.022	−0.252	0.801
	ABS_EXP_mean_T0 ⇒ MPA_T1	0.695	0.418	−0.124	1.515	0.154	1.662	0.096
	MPA_T1 ⇒ ACT_T1	−0.091	0.098	−0.283	0.101	−0.080	−0.929	0.353
Direct	ABS_EXP_mean_T0 ⇒ ACT_T1	−1.471	0.460	−2.373	−0.570	−0.287	−3.199	0.001
Total	ABS_EXP_mean_T0 ⇒ ACT_T1	−1.962	0.438	−2.820	−1.104	−0.377	−4.482	< 0.001

Model 2								
Indirect and total effects								
				95% CI (a)				
Type	Effect	Estimate	SE	Lower	Upper	*β*	*z*	*p*
Indirect	IAT_T0 ⇒ NA_T1 ⇒ ACT_T1	0.515	0.353	−0.177	1.206	0.048	1.459	0.144
	IAT_T0 ⇒ GHP_T1 ⇒ ACT_T1	−0.073	0.142	−0.351	0.204	−0.007	−0.518	0.604
	IAT_T0 ⇒ MPA_T1 ⇒ ACT_T1	0.078	0.126	−0.169	0.324	0.007	0.616	0.538
Component	IAT_T0 ⇒ NA_T1	−0.411	0.246	−0.892	0.070	−0.161	−1.673	0.094
	NA_T1 ⇒ ACT_T1	−1.252	0.420	−2.075	−0.429	−0.295	−2.983	0.003
	IAT_T0 ⇒ GHP_T1	4.118	3.097	−1.952	10.187	0.129	1.330	0.184
	GHP_T1 ⇒ ACT_T1	−0.018	0.032	−0.080	0.044	−0.053	−0.563	0.574
	IAT_T0 ⇒ MPA_T1	−0.830	0.926	−2.645	0.984	−0.087	−0.897	0.370
	MPA_T1 ⇒ ACT_T1	−0.093	0.110	−0.309	0.122	−0.082	−0.849	0.396
Direct	IAT_T0 ⇒ ACT_T1	1.418	1.000	−0.542	3.378	0.131	1.418	0.156
Total	IAT_T0 ⇒ ACT_T1	2.002	0.987	0.068	3.937	0.188	2.029	0.043

Model 3								
Indirect and total effects								
				95% CI (a)				
Type	Effect	Estimate	SE	Lower	Upper	*β*	*z*	*p*
Indirect	IMP_WOR_mean_T0 ⇒ NA_T1 ⇒ DeltaACT	0.002	0.016	−0.030	0.033	0.000	0.099	0.921
	IMP_WOR_mean_T0 ⇒ GHP_T1 ⇒ DeltaACT	0.001	0.044	−0.086	0.087	0.000	0.018	0.986
	IMP_WOR_mean_T0 ⇒ MPA_T1 ⇒ DeltaACT	0.010	0.030	−0.048	0.068	0.002	0.331	0.741
Component	IMP_WOR_mean_T0 ⇒ NA_T1	0.014	0.136	−0.252	0.280	0.010	0.103	0.918
	NA_T1 ⇒ DeltaACT	0.112	0.338	−0.551	0.775	0.033	0.332	0.740
	IMP_WOR_mean_T0 ⇒ GHP_T1	−1.638	1.674	−4.919	1.643	−0.091	−0.979	0.328
	GHP_T1 ⇒ DeltaACT	−0.000	0.027	−0.053	0.052	−0.002	−0.018	0.986
	IMP_WOR_mean_T0 ⇒ MPA_T1	0.239	0.487	−0.716	1.194	0.046	0.491	0.623
	MPA_T1 ⇒ DeltaACT	0.041	0.091	−0.138	0.220	0.042	0.447	0.655
Direct	IMP_WOR_mean_T0 ⇒ DeltaACT	−0.723	0.466	−1.636	0.191	−0.144	−1.551	0.121
Total	IMP_WOR_mean_T0 ⇒ DeltaACT	−0.803	0.451	−1.687	0.081	−0.160	−1.780	0.075

*Notes:* Confidence intervals computed with method: Standard (*Δ* method). *β* values are completely standardised effect sizes.

Abbreviations: ABS = Absolute Expectations, ACT = Asthma Control Test, Delta, ACT = difference between ACT score at T0 and T1, GHP = Good Health Practices, HADS‐A = HADS‐Anxiety, HADS‐D = HADS‐Depression, IAT = IAT‐IE, IMP_WOR = Improvement/Worsening, MPA = Measures of Patient Adherence, NA = Negative Affects, PSS = Perceived Stress Scale, RIG = Rigidity.

## 4 Discussion

The investigation into the role of IEs in predicting disease‐related outcomes, thanks to the implementation of a longitudinal cohort study, yielded notable findings. First, ABS emerged as a significant predictor for both ACT and FVC outcomes, even when emotional factors and illness‐related behaviours were considered. Second, the IMP factor strongly predicted ACT scores, while not influencing FVC values over time, even after adjusting for confounders. While ACT is a validated and widely used self‐report tool for evaluating asthma control, in this study, it was not a clinical endpoint per se, but rather one of several outcome variables used to assess the predictive validity of IEs. The associations we observed point to a possible contribution of expectation‐related cognitive processes in shaping illness trajectories, but should be interpreted cautiously, given the observational design and sample size. Importantly, although the explained variance was modest, the predictive effect of explicit expectations remained significant even after accounting for known psychosocial and behavioural factors.

Conversely, implicit IEs failed to show significant associations with disease‐related outcomes. The lack of significant findings for implicit IEs should not be taken to imply irrelevance. Implicit processes, by nature, are difficult to capture through current measurement tools and may exert subtler effects on health outcomes. Future studies using alternative assessment strategies (e.g., implicit association paradigms or neurocognitive probes) could help clarify their role. It is also possible that implicit and explicit expectations act through different mechanisms or at different stages of illness management, a hypothesis that warrants further investigation.

From a clinical perspective, our results tentatively support the value of considering patients' expectations during asthma management. Recognising the way patients think about their illness—particularly their expectations regarding symptoms and progression—may provide insight into their health‐related behaviour and treatment engagement. While not yet ready for routine screening or decision‐making, brief tools such as those used in this study could, in future, support more individualised approaches to care by identifying patients with particularly negative or maladaptive expectations.

Our findings resonate with research in other domains, where expectations have been shown to influence symptom perception and treatment response. While previous research underscores the significant impact of the placebo effect on a neurobiological level, influencing immune, neuroendocrine and autonomic nervous system responses [59, 60, 61], IE extends beyond placebo by encompassing patients' cognitive frameworks formed after diagnosis, independently shaping symptom perception and health trajectories [23]. IE, however, is a comprehensive idea that goes well beyond the placebo effect, albeit offering a potential explanation for it. Individuals with IEs arise on their own, and those who are medically diagnosed will soon begin to form assumptions about their conditions [62, 63]. Therefore, there is a need to explore the potential effects of IEs as a form of placebo, further delving into their impact within this context.

Third, in a more specific examination of the impact of ABS in a mediation model on medical variables, the results highlighted its significant direct effect on ACT scores, indicating a worsening impact on symptomatology with increasing ABS. The effects of ABS on ACT, mediated by NA, such as stress, anxiety and depression, were also significant. In contrast, no significant direct effect of IE‐IAT on ACT scores was observed. While implicit IEs did not reach statistical significance and demonstrated a small effect size, it remains important to consider their potential role in illness perception. Future studies with larger samples and alternative measures may help clarify their relevance. The lack of significant results for implicit IEs may reflect methodological challenges, such as the limited test–retest reliability of tools designed to measure unconscious expectations or the difficulty of capturing nuanced, subconscious processes with existing methods. Alternatively, it could indicate that implicit IEs exert a weaker or more indirect influence on asthma outcomes compared to explicit IEs. Additionally, the relationship between IEs and asthma outcomes may be bidirectional, with negative outcomes reinforcing pessimistic expectations, creating a self‐perpetuating cycle. Future studies should explore this dynamic further, using longitudinal designs to disentangle causality and refining tools for implicit IEs assessment. Moreover, IMP of the illness did not demonstrate a significant direct effect on the change in ACT scores between the considered time points. Its impact on ACT scores, mediated by stress, anxiety and depression (NA), adopted behaviours (GHP), and medication adherence (MPA), similarly yielded non‐significant effects. These findings reaffirm the significance of viewing the patient as an integral participant in the care process. The outdated notion of a passive patient solely reliant on treatment and medical opinions, neglecting the role of patient engagement, needs to be discarded [64]. Patients should be empowered to take charge of their care, recognising that symptoms can evolve over time, influenced in part by their attitudes toward them [65, 66]. Conversely, healthcare providers need to be aware that their communication with patients can elicit diverse reactions [67], subsequently influencing changes in the progression of the disease [68]. For instance, conveying a negative perspective can steer the patient's expectations towards unfavourable outcomes, with foreseeable negative impacts over time [69, 70]. This underscores the importance of incorporating awareness in clinical training regarding how healthcare professionals induce expectations.

### 4.1 Strengths and Limitations

This study offers a comprehensive and innovative approach to examining IEs in asthma, addressing both explicit and implicit expectations. This dual perspective provides a richer understanding of the cognitive patterns that influence disease management. The longitudinal design, with three assessments over a 6‐month period, allows us to examine changes over time, providing valuable insights into the relationship between disease expectations and asthma symptoms. Furthermore, the study incorporates objective measures, such as spirometry, which contribute to the robustness of the findings, despite the challenges faced during data collection. The advanced statistical methods employed, including LMM and GLM, enhance the reliability and validity of the results, and the use of a previously validated instrument to assess IEs strengthens the credibility of our measurements.

Several significant limitations must be acknowledged that may affect the interpretation and generalisability of our findings.

1. *Missing Data and Dropout Rates*: A key limitation of this study is the high dropout rate and missing spirometry data, largely due to logistical constraints imposed by the SARS‐CoV‐2 pandemic. These disruptions limited the availability of objective respiratory function measures, narrowing the scope of our analysis. Despite efforts to mitigate bias by excluding variables with high missing values from our GLMs, these missing data could still influence the generalisability of the results. Participants who dropped out or were unable to complete spirometry assessments may differ systematically from those who completed the study, potentially impacting the observed relationships between IEs and asthma outcomes. Future research should consider using digital health tools for remote assessments to reduce dropout rates and improve data completeness.
2. *Self‐Reported Data*: Our reliance on self‐reported asthma symptoms, particularly through the ACT, introduces potential biases, such as recall bias and variability in symptom interpretation. Emotional and psychological factors, particularly during the pandemic, may have led to either overreporting or underreporting of symptoms. While the ACT offers a valuable patient perspective, these biases emphasise the need for complementary objective measures, such as spirometry, to accurately capture asthma symptom severity.
3. *Scope and Applicability*: The study's focus on asthma patients and specific assessment tools limits the broader applicability of the findings to other chronic diseases or general patient populations. Future studies should aim to validate these findings in diverse clinical settings and explore the role of IEs in other chronic conditions to enhance the generalisability of the results.
4. *Lack of Statistical Significance for Implicit IEs*: The lack of statistical significance for implicit IEs in this study limits the strength of the evidence supporting their predictive capacity. While explicit IEs showed a significant relationship with asthma symptoms and respiratory function, implicit IEs' role remains uncertain and warrants further investigation. Future studies with larger cohorts and more refined measurement tools could help clarify the potential impact of implicit IEs on disease outcomes.
5. *Study Protocol and Transparency*: The absence of pre‐registration of the study protocol is another limitation, as it may affect the transparency and replicability of our findings. Pre‐registration would have provided clarity on the hypotheses and methodology, ensuring a rigorous definition of the study design from the outset.

### 4.2 Future Directions

Despite these limitations, our findings underscore the potential importance of explicit IEs in asthma management. Moving forward, future research should address the identified limitations, particularly the high dropout rates and reliance on self‐reported data. Incorporating socio‐demographic factors, including healthcare access and socioeconomic status, would provide a more comprehensive understanding of how IEs interact with asthma outcomes. Additionally, a deeper exploration of implicit IEs could refine intervention strategies.

Integrating digital health tools to monitor both explicit and implicit IEs in real time could improve symptom management and lead to more personalised, patient‐centred interventions [71].

### 4.3 Clinical and Research Implications

The findings highlight the predictive value of explicit expectations in relation to asthma symptom progression, with optimistic expectations associated with clinical improvements and negative expectations linked to worsening symptoms over time. These insights suggest that fostering positive expectations in patients could be a useful strategy in asthma care, potentially improving treatment adherence and symptom control. Moreover, in the routine management of asthma, it might be relevant to use tools such as those we used in the study to make brief assessments of IEs during clinical consultations.

Future research can also explore the applicability of the IET in different healthcare contexts and/or in countries of different cultures. Furthermore, recognising the role of IEs in asthma progression emphasises the importance of open and effective communication between patients and healthcare providers. While the study focused on asthma, comparative research across various chronic diseases (e.g., chronic obstructive pulmonary disease, diabetes or cardiovascular disease) could shed light on the generalisability of the findings. Exploring similarities and differences in the role of IEs across conditions could contribute to broader healthcare understanding. Moreover, addressing IEs in these conditions could similarly enhance self‐management, treatment adherence and overall outcomes, underscoring the broader relevance of these insights. Future research can delve deeper into understanding how these expectations evolve over extended periods and their sustained influence on health outcomes.

## 5 Conclusions

This study confirms the prognostic value of IEs in predicting asthma symptom progression. Specifically, explicit expectations were strongly associated with symptom outcomes, with optimistic expectations linked to clinical improvements and negative expectations predicting worsening symptoms. Although implicit expectations yielded inconsistent results, the role of explicit IEs—particularly those regarding anticipated symptoms—was clear in forecasting symptom severity.

The reliance on self‐reported data provided valuable insights into the relationship between patient expectations and symptom severity, though the high dropout rate and missing objective data due to pandemic‐related constraints limit the robustness of these findings. Despite these limitations, a statistically significant and clinically meaningful relationship between expectations and the severity of symptoms was observed. Future research is needed to confirm these results and to explore the utility of addressing IEs in improving asthma management and patient outcomes.

## Author Contributions

**Eleonora Volpato:** conceptualisation, writing – original draft, writing – review and editing, validation, methodology, visualisation, software, data curation, project administration, investigation. **Valentina Poletti:** methodology, investigation. **Paolo Banfi:** investigation, methodology. **Andrea Bonanomi:** software, formal analysis, data curation. **Francesco Pagnini:** conceptualisation, investigation, methodology, funding acquisition, writing – review and editing, supervision.

## Ethics Statement

The project was carried out in accordance with the Helsinki Declaration, and the protocol was authorised by the Università Cattolica del Sacro Cuore's Ethics Committee (cod. 35–18, 21/12/2018), in Milan, Italy, and by the IRCCS Fondazione Don Carlo Gnocchi's Ethics Committee (cod. 14/2019/CE_FdG/FC/SA, 17/04/2019).

## Consent

In accordance with Italian Law 196/2003 on Privacy and Safeguarding of Sensitive Data and the GDPR of the European Union 2016/679, the consent form was shared before the interview. The dissemination of the participants' anonymised responses was permitted with their informed consent.

## Conflicts of Interest

Professor Francesco Pagnini reports grants from the Bial Foundation during the conduct of the study. The authors reported no other potential conflicts of interest for this study.

## Supporting information

Supporting Material 1: Strengthening the Reporting of Observational Studies in Epidemiology (STROBE) Checklist.

## Data Availability

The datasets used and/or analysed during the current study are available from the corresponding author upon reasonable request.
