# Supplementary material for: Illness Expectations and Asthma Symptoms: A 6‐Month Longitudinal Study
Source: Health Expect. 2025 May 5;28(3):e70285. doi: 10.1111/hex.70285 (PMC12053105; doi:10.1111/hex.70285)
Supplement: Supplementary file 1 — Supporting Material 1: Strengthening the Reporting of Observational Studies in Epidemiology (STROBE) Checklist. [file HEX-28-e70285-s001.doc]

STROBE Statement—checklist of items that should be included in reports of observational studies

|  | Item No | Recommendation |
| --- | --- | --- |
| **Title and abstract** | 1 | (*a*) Indicate the study’s design with a commonly used term in the title or the abstract Page 1 |
|  |  | (*b*) Provide in the abstract an informative and balanced summary of what was done and what was found Page 2 |
| Introduction | | |
| Background/rationale | 2 | Explain the scientific background and rationale for the investigation being reported Pages 3-6 |
| Objectives | 3 | State specific objectives, including any prespecified hypotheses Page 7 |
| Methods | | |
| Study design | 4 | Present key elements of study design early in the paper Pages 9-10 |
| Setting | 5 | Describe the setting, locations, and relevant dates, including periods of recruitment, exposure, follow-up, and data collection Pages 9-10 |
| Participants | 6 | (*a*) *Cohort study*—Give the eligibility criteria, and the sources and methods of selection of participants. Describe methods of follow-up  *Case-control study*—Give the eligibility criteria, and the sources and methods of case ascertainment and control selection. Give the rationale for the choice of cases and controls  *Cross-sectional study*—Give the eligibility criteria, and the sources and methods of selection of participants Page 8 |
|  |  | (*b*) *Cohort study*—For matched studies, give matching criteria and number of exposed and unexposed  *Case-control study*—For matched studies, give matching criteria and the number of controls per case |
| Variables | 7 | Clearly define all outcomes, exposures, predictors, potential confounders, and effect modifiers. Give diagnostic criteria, if applicable Pages 10-13 |
| Data sources/ measurement | 8* | For each variable of interest, give sources of data and details of methods of assessment (measurement). Describe comparability of assessment methods if there is more than one group Pages 10-13 |
| Bias | 9 | Describe any efforts to address potential sources of bias Page 15 |
| Study size | 10 | Explain how the study size was arrived at Pages 9-10 |
| Quantitative variables | 11 | Explain how quantitative variables were handled in the analyses. If applicable, describe which groupings were chosen and why Pages 14-16 |
| Statistical methods | 12 | (*a*) Describe all statistical methods, including those used to control for confounding Pages 13-14 |
|  |  | (*b*) Describe any methods used to examine subgroups and interactions Pages 13-15 |
|  |  | (*c*) Explain how missing data were addressed Pages 13-15, 20 |
|  |  | (*d*) *Cohort study*—If applicable, explain how loss to follow-up was addressed  *Case-control study*—If applicable, explain how matching of cases and controls was addressed  *Cross-sectional study*—If applicable, describe analytical methods taking account of sampling strategy Pages 13-15 |
|  |  | (*e*) Describe any sensitivity analyses Pages 13-15 |

Continued on next page

| Results | | |
| --- | --- | --- |
| Participants | 13* | (a) Report numbers of individuals at each stage of study—eg numbers potentially eligible, examined for eligibility, confirmed eligible, included in the study, completing follow-up, and analysed Pages 15-16 |
|  |  | (b) Give reasons for non-participation at each stage Pages 15-16 |
|  |  | (c) Consider use of a flow diagram Figure 1 |
| Descriptive data | 14* | (a) Give characteristics of study participants (eg demographic, clinical, social) and information on exposures and potential confounders Pages 15-16; Figure 1; Table 1 |
|  |  | (b) Indicate number of participants with missing data for each variable of interest Pages 15-16; Figure 1; Table 1 |
|  |  | (c) *Cohort study*—Summarise follow-up time (eg, average and total amount) Pages 15-16; Figure 1; Table 1 |
| Outcome data | 15* | *Cohort study*—Report numbers of outcome events or summary measures over time Page 8 |
|  |  | *Case-control study—*Report numbers in each exposure category, or summary measures of exposure N/A |
|  |  | *Cross-sectional study—*Report numbers of outcome events or summary measures N/A |
| Main results | 16 | (*a*) Give unadjusted estimates and, if applicable, confounder-adjusted estimates and their precision (eg, 95% confidence interval). Make clear which confounders were adjusted for and why they were included Pages 15-17; Table 2; Table 3; Figure 2; Figure 3 |
|  |  | (*b*) Report category boundaries when continuous variables were categorized Pages 15-17; Table 2; Table 3; Figure 2; Figure 3 |
|  |  | (*c*) If relevant, consider translating estimates of relative risk into absolute risk for a meaningful time period Pages 15-17; Table 2; Table 3; Figure 2; Figure 3 |
| Other analyses | 17 | Report other analyses done—eg analyses of subgroups and interactions, and sensitivity analyses Pages 15-17; Table 2; Table 3; Figure 2; Figure 3; Figure 4 |
| Discussion | | |
| Key results | 18 | Summarise key results with reference to study objectives Pages 17-22 |
| Limitations | 19 | Discuss limitations of the study, taking into account sources of potential bias or imprecision. Discuss both direction and magnitude of any potential bias Pages 20-22 |
| Interpretation | 20 | Give a cautious overall interpretation of results considering objectives, limitations, multiplicity of analyses, results from similar studies, and other relevant evidence Pages 17-23 |
| Generalisability | 21 | Discuss the generalisability (external validity) of the study results Pages 22-23 |
| Other information | | |
| Funding | 22 | Give the source of funding and the role of the funders for the present study and, if applicable, for the original study on which the present article is based Mentioned following the indication of the journal |

*Give information separately for cases and controls in case-control studies and, if applicable, for exposed and unexposed groups in cohort and cross-sectional studies.

**Note:** An Explanation and Elaboration article discusses each checklist item and gives methodological background and published examples of transparent reporting. The STROBE checklist is best used in conjunction with this article (freely available on the Web sites of PLoS Medicine at http://www.plosmedicine.org/, Annals of Internal Medicine at http://www.annals.org/, and Epidemiology at http://www.epidem.com/). Information on the STROBE Initiative is available at www.strobe-statement.org.
